# Pancreas in the chest: Diaphragmatic hernia with obstruction

**DOI:** 10.1016/j.radcr.2025.09.006

**Published:** 2025-09-20

**Authors:** Jawdat M. Alali, Ahmad L.F. Yasin, Mohammad Odeh, Mahmoud Tabouni, Nissar M. Shaikh

**Affiliations:** a Surgical Intensive Care Unit, Hamad Medical Corporation (HMC), Doha, Qatar; b Dept of Interventional Radiology, Hamad Medical Corporation, Doha, Qatar; c Pediatric Pulmonology Dept, Sidra Medicine, Doha, Qatar; d Internal Medicine Dept, Hamad Medical Corporation (HMC), Doha, Qatar

**Keywords:** Pancreatic herniation, Diaphragmatic hernia, Colonic obstruction, Congenital defect, Elderly, Thoracoabdominal CT

## Abstract

Diaphragmatic hernias involving abdominal viscera are uncommon in adults and rarely involve the pancreas. While congenital diaphragmatic hernias (CDH) typically present in infancy, late presentations may occur with non-specific gastrointestinal or respiratory symptoms, often leading to delayed diagnosis. Cross-sectional imaging plays a pivotal role in identifying the extent and contents of herniation, especially in complex or atypical cases.

We present a case of A 91-year-old woman with known rectosigmoid adenocarcinoma who presented with abdominal pain, constipation, nausea, and persistent cough. Chest X-ray revealed cystic lucencies within the right hemithorax, associated with blurring of the right hemidiaphragm and relative paucity of bowel gas in the upper abdomen. These findings were concerning for an underlying structural abnormality of the diaphragm. Thoraco-abdominal CT demonstrated a large right diaphragmatic hernia containing the stomach, colonic loops, the body and tail of the pancreas, and mesenteric fat and vessels. Additional findings included colonic dilation and peritoneal free fluid, consistent with large bowel obstruction secondary to malignancy. No history of trauma was reported, favoring a congenital etiology.

This case highlights an unusual radiological finding of pancreatic herniation into the thorax through a right-sided diaphragmatic defect. It emphasizes the importance of cross-sectional imaging in diagnosing complex hernias, particularly in elderly patients with overlapping thoracoabdominal symptoms.

## Introduction

Diaphragmatic hernias are characterized by the abnormal migration of abdominal contents into the thoracic cavity through a defect in the diaphragm. These hernias are typically classified as congenital or acquired, with acquired causes most commonly resulting from trauma or surgical interventions. However, in elderly individuals, spontaneous herniation may occur due to progressive weakening of diaphragmatic musculature with age [1,2].

Among the types of diaphragmatic hernias, Type IV hiatal hernias are the most severe, involving herniation of multiple abdominal organs, including the stomach, colon, omentum, and, very rarely, the pancreas [3,4]. Pancreatic herniation is especially unusual due to the retroperitoneal location and tethering of the organ by vascular and ligamentous structures. It is typically reported in the context of large paraesophageal hernias or traumatic diaphragmatic defects. Here, we present an exceptional case of a large right-sided diaphragmatic hernia in a non-traumatized 91-year-old woman, which included herniation of the body and tail of the pancreas, along with bowel loops and mesenteric structures, incidentally discovered during the evaluation of abdominal pain and bowel obstruction in the setting of colorectal malignancy.

## Case presentation

A 91-year-old woman presented to the emergency department with a 4-day history of progressively worsening abdominal pain, persistent cough, constipation, nausea, and a single episode of vomiting. Her medical history was significant for rectosigmoid adenocarcinoma, previously diagnosed as causing rectosigmoid intussusception and confirmed to be a moderately differentiated invasive adenocarcinoma. Additional comorbidities included hypothyroidism (managed with levothyroxine), bronchial asthma, dyslipidemia (on simvastatin), and chronic anemia. There was no history of trauma or prior thoracoabdominal surgery.

On physical examination, the patient was alert and oriented with a Glasgow Coma Scale (GCS) score of 15. Her vital signs were within normal limits, and she was hemodynamically stable. On admission screening, she tested positive for COVID-19, though her respiratory status was stable and she did not require supplemental oxygen.

**Fig. 1:**
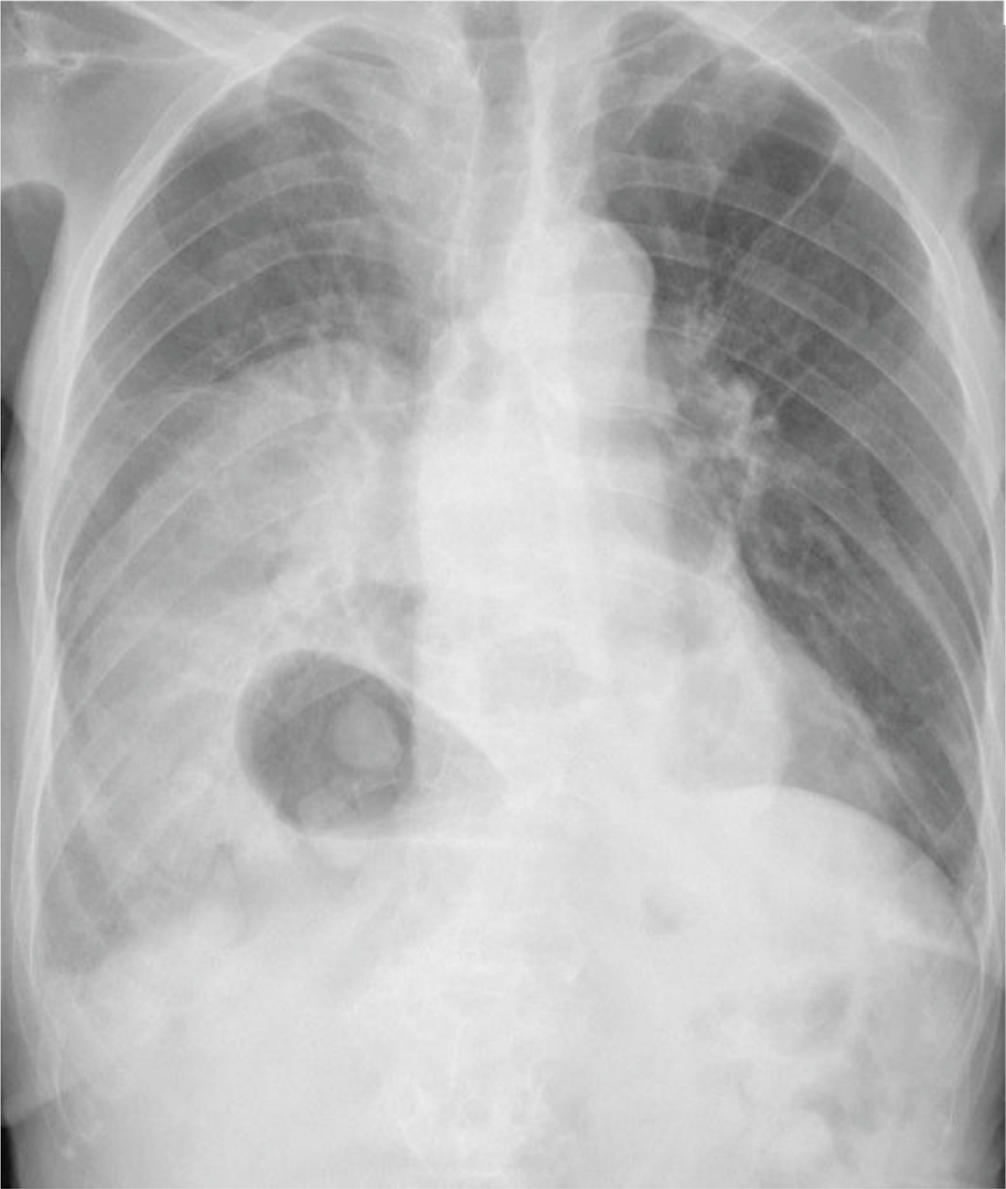
Frontal chest X-ray demonstrates multiple cystic lucencies within the right hemithorax, with an indistinct right hemidiaphragm and relative paucity of bowel gas in the upper abdomen. The left lung field appears clear.

A chest X-ray (Fig. 1) was obtained to evaluate her persistent cough. The imaging demonstrated new cystic lucencies within the right hemithorax, associated with blurring of the right hemidiaphragm and relative paucity of bowel gas in the upper abdomen. The left lung field appeared unremarkable. These findings were concerning for an underlying structural abnormality of the diaphragm.

**Fig. 2:**
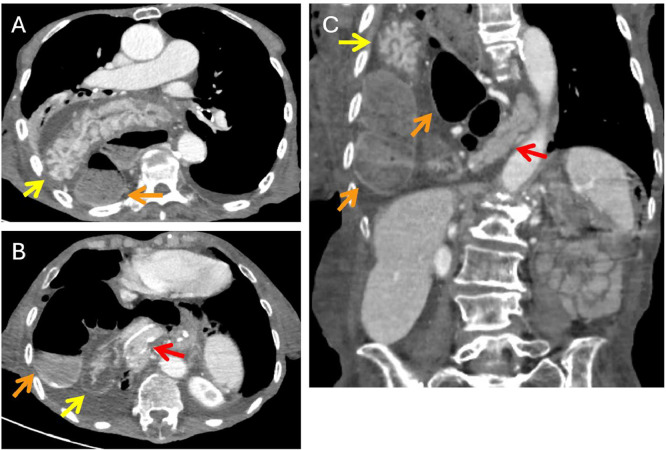
Axial (A, B) and coronal reconstruction (C) of thoraco-abdominal CT scan show herniated abdominal content including stomach (yellow arrows), colonic loops (orange arrows) and body and tail of the pancreas (red arrows) along with mesenteric fat and vessels consistent with diaphragmatic hernia. Note the dilated colonic bowel loops and the presence of free fluid related to chronic distal large bowel obstruction.

To further evaluate the findings, a contrast-enhanced thoraco-abdominal computed tomography (CT) scan was performed. The CT scan, including both axial (Fig. 2A, B) and coronal (Fig. 2C) reconstructions, confirmed the presence of a large right diaphragmatic hernia. Several abdominal structures were noted to have herniated into the right thoracic cavity. These included the stomach (noted with yellow arrows), segments of the colon (orange arrows), and notably, the body and tail of the pancreas (red arrows). Associated mesenteric fat and vascular structures were also visualized within the hernia sac. The herniation resulted in a mild mediastinal shift to the left. The chronic nature of the herniated organs and absence of any traumatic or iatrogenic insult suggested a congenital etiology for the diaphragmatic defect, which had gone undiagnosed until this presentation.

In addition to the herniation, the CT scan revealed markedly dilated colonic loops and the presence of free peritoneal fluid, findings consistent with a large bowel obstruction. The obstruction was attributed to progression of the known rectosigmoid malignancy.

Given the obstructive symptoms and the underlying rectosigmoid cancer, the patient underwent urgent flexible sigmoidoscopy under sedation. A self-expanding metallic colonic stent was successfully deployed to relieve the malignant obstruction. Post-procedure, the patient was managed conservatively. She was started on oral clear fluids and gradually advanced her diet. She was able to pass stool on the second postoperative day, indicating relief of the obstruction.

Considering her advanced age, multiple comorbidities, and the absence of acute signs of strangulation or perforation, a non-operative approach was favored for the management of the diaphragmatic hernia. The patient remained clinically stable with no respiratory compromise, and conservative monitoring was continued.

## Discussion

This case highlights a rare radiological finding of pancreatic herniation into the thoracic cavity in an elderly patient without a history of trauma or prior thoracoabdominal surgery. The presence of the pancreas within a right-sided diaphragmatic hernia is exceptionally uncommon and suggests a congenital etiology, especially in the absence of acute inciting events and the chronic appearance of the herniated contents on imaging.

Pancreatic herniation is most often associated with large Type IV hiatal hernias, which represent advanced herniations of intra-abdominal contents through the esophageal hiatus [1,4]. In such cases, herniated organs may include the colon, spleen, and small bowel, but pancreatic involvement remains rare due to its relatively fixed retroperitoneal position. When pancreatic herniation does occur, it carries potential risks such as vascular compromise, torsion, or pancreatitis [4].

Hiatal and diaphragmatic hernias increase in prevalence with age, likely due to age-related weakening of the diaphragmatic crura and widening of the hiatus [2]. Other risk factors include female sex, obesity, and chronic increases in intra-abdominal pressure [3]. Although diaphragmatic hernias may remain asymptomatic, they can manifest with a spectrum of symptoms—ranging from respiratory complaints like cough or dyspnea to gastrointestinal issues such as reflux, nausea, or obstructive symptoms. In this patient, a persistent cough and signs of bowel obstruction likely reflected the combined effects of the hernia and her underlying rectosigmoid adenocarcinoma.

Imaging played a crucial role in establishing the diagnosis. The frontal chest X-ray revealed multiple air-filled bowel loops within the right hemithorax and an indistinct right hemidiaphragm, raising suspicion of herniation. Thoraco-abdominal CT confirmed the presence of a large diaphragmatic defect with herniation of the stomach, colon, pancreas, mesenteric fat, and vessels. These findings are essential not only for diagnosis but also for surgical planning and risk assessment.

## Conclusion

This case illustrates a rare and dramatic occurrence of pancreatic herniation through a right-sided diaphragmatic defect in an elderly patient with colorectal cancer. In the absence of trauma or surgery, a congenital etiology is likely. The presence of abdominal symptoms and obstructive features, in combination with unusual radiographic findings, underscores the importance of considering diaphragmatic hernia in the differential diagnosis, particularly when bowel gas is visualized in the thoracic cavity. Cross-sectional imaging, especially CT, is invaluable for identifying the contents of the hernia and potential complications. Clinicians should maintain a high index of suspicion for such rare presentations to facilitate timely diagnosis and management.

## Patient consent

Written, informed consent was obtained from the patient for publication of this case report and accompanying images.
